# Graphene Oxide and Conductive Polymer–Enhanced Langmuir–Blodgett Biosensor for Sensitive Detection of Pyrocatechol

**DOI:** 10.1021/acsomega.5c11698

**Published:** 2026-02-16

**Authors:** Felipe Merloto Marinho, Coral Salvo-Comino, Maria Luz Rodriguez-Mendez, José Roberto Siqueira Junior, Luciano Caseli

**Affiliations:** † Hybrid Materials Laboratory, Department of Chemistry, Institute of Environmental, Chemical and Pharmaceutical Sciences, Federal University of São Paulo, Diadema 09913-030, Brazil; ‡ Group UVASENS, Department of Inorganic Chemistry. Escuela de Ingenierías Industriales, 16782 University of Valladolid, Valladolid 47011, Spain; § BioecoUVA Research Institute, University of Valladolid, Valladolid 47001, Spain; ∥ Laboratory of Applied Nanomaterials and Nanostructures, Department of Physics, Institute of Exact and Natural Science and Education, Federal University of Triângulo Mineiro, Uberaba 38.064-200, Brazil

## Abstract

In this work, we report a novel Langmuir–Blodgett (LB) biosensing platform for the detection of phenolic compounds, using Pyrocatechol as a model analyte. Although LB films have long been explored for polyphenol detection, the present study introduces an innovative hybrid architecture that integrates a lipid matrix (DMPA), a conductive polymer (P3HT), graphene oxide (GO), and laccase into a single, highly organized ultrathin film. This configuration simultaneously enhances film rigidity, reduces surface roughness, and modulates electron-transfer properties in a way not previously reported for LB-based enzymatic sensors. Comprehensive interfacial characterization (surface pressure–area isotherms, dilatational rheology, UV–Vis, AFM) reveals that GO plays a decisive role in promoting compact molecular packing and stabilizing the enzyme–polymer–lipid assembly. As a consequence, the resulting LB films exhibit significantly improved electrochemical performance, including nearly 2-fold higher sensitivity, lower detection limits, and reduced overpotentials compared with films lacking GO. The study also provides mechanistic evidence that the synergy between conductive polymer domains, GO nanosheets, and the immobilized enzyme facilitates more efficient redox cycling of Pyrocatechol. These findings demonstrate that the rational incorporation of GO into LB enzymatic architectures offers a promising route toward next-generation ultrathin biosensors with enhanced analytical performance and structural stability.

## Graphical abstract

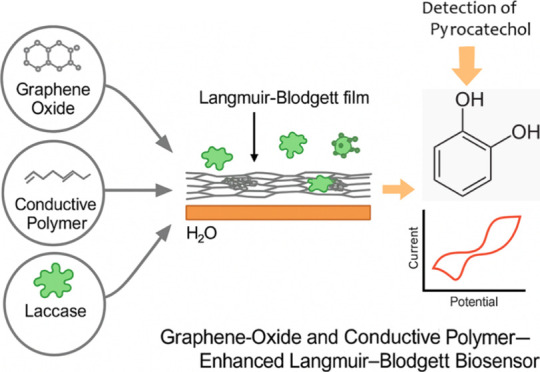

## 1 Introduction

Polyphenols are a broad class of compounds widely distributed in foods, beverages, and natural waters, where they strongly influence flavor, color, stability, and antioxidant activity.^1–4^ Their presence is also relevant to environmental monitoring, as many phenolics are industrial pollutants with toxicological implications.^5–7^ For these reasons, the development of rapid, cost-effective, and reliable methods for the detection and quantification of phenolic compounds has attracted increasing attention.^8–10^

Enzyme-based biosensors, particularly those employing laccases, have emerged as promising candidates due to their high specificity and catalytic efficiency.^11–13^ Laccases are blue multicopper oxidases found in plants, fungi, and some microorganisms, capable of catalyzing the oxidation of a wide variety of phenolics.^14–16^ Their catalytic site features a type 1 copper center (T1) responsible for substrate oxidation and a trinuclear T2/T3 cluster that reduces molecular oxygen to water, making them highly effective biocatalysts for phenolic detection. Owing to these properties, laccases are increasingly explored in sensor platforms for food analysis, environmental monitoring, and industrial process control.

A key challenge for laccase-based biosensors is achieving efficient enzyme immobilization without compromising catalytic activity. Langmuir and Langmuir–Blodgett (LB) films provide a versatile and biomimetic strategy for this purpose, offering ordered molecular architectures that preserve enzymatic functionality and allow precise integration with conductive and nanostructured materials.^17–20^ In addition, the incorporation of conducting polymers into these structures has attracted interest, as it enhances sensing performance and facilitates electron transfer.^21–26^

Recently, our group demonstrated that immobilizing laccase in LB films of octadecylamine (ODA) and the conductive polymer poly­(3-hexylthiophene-2,5-diyl) (P3HT) enhanced the sensitivity of biosensors toward mono-, di-, and triphenols, evidencing how organized nanostructures can boost enzyme activity and electrochemical performance.^27^ Building on these findings, we now incorporate graphene oxide (GO) to further improve film rigidity, reduce hysteresis, and modulate electron transfer processes.

The incorporation of graphene oxide and conductive polymers into ultrathin films has proven particularly advantageous for biosensing applications. Graphene oxide provides a large surface area, abundant oxygenated groups for enzyme anchoring, and mechanical reinforcement, which together improve film stability and sensitivity.^28–30^ In parallel, conjugated polymers such as poly­(3-hexylthiophene-2,5-diyl) (P3HT) act as electron mediators, facilitating charge transfer between the biocatalyst and the electrode while maintaining environmental stability.^23,31,32^ When integrated in organized Langmuir–Blodgett architectures, these components synergistically enhance catalytic performance, lower detection limits, and enable the design of highly reproducible ultrathin biosensors tailored for environmental and industrial monitoring.^33–37^

In this study, we report the development of nanostructured LB films composed of 1,2-dimyristoyl-*sn*-glycero-3-phosphate (sodium salt) (DMPA), P3HT, GO, and laccase from *Trametes versicolor*. The system was investigated at the air–water interface and transferred onto solid supports to fabricate biosensors. Pyrocatechol was selected as a model analyte to demonstrate the applicability of the films for phenolic compound detection. The incorporation of GO and P3HT played a crucial role in modulating film rigidity, conductivity, and catalytic response. The primary objective of this work is to evaluate the structural, physicochemical, and electrochemical properties of these hybrid LB films and to demonstrate their potential as sustainable biosensing platforms for environmental and industrial applications.

## 2 Materials and Methods

### 2.1 Chemicals

All reagents were of analytical grade and purchased from Sigma-Aldrich (St. Louis, MO, USA). Solutions were prepared using Milli-Q deionized water (Merck KGaA, Darmstadt, Germany). Chloroform was obtained from Sigma-Aldrich. Indium tin oxide (ITO) glass was acquired from Merck. The lipid 1,2-dimyristoyl-*sn*-glycero-3-phosphate (sodium salt) (DMPA) was obtained from Avanti Polar Lipids, while the conductive polymer poly­(3-hexylthiophene-2,5-diyl) (P3HT), graphene oxide (GO), Laccase from *T. versicolor* and the phenolic compounds, including Pyrocatechol, were purchased from Sigma-Aldrich.

Laccase was dispersed in 0.1 M phosphate buffer (pH 7.4) to a concentration of 1.0 mg/mL and GO in water to prepare a suspension of 4.4 mg/mL. DMPA and P3HT were each one dissolved in chloroform (CHCl_3_) at 0.5 mg/mL.

The graphene oxide (GO) used in this study was supplied in powder form. According to the manufacturer, the material consists of 15–20 stacked layers, with an edge-oxidation degree of 4–10%, and an average flake thickness corresponding to 15–20 sheets. This GO is characterized by its high dispersibility in water and other polar solvents, which facilitates its exfoliation and incorporation into Langmuir–Blodgett films. Prior to the utilization in the Langmuir monolayers, it was dispersed in water for a concentration of 1 mg/mL.

Water was purified using a Milli-Q system from Millipore, achieving a resistivity of 18.2 MΩ·cm, and chloroform was obtained from LabSynth. The temperature was maintained at 25 ± 1 °C for all experiments.

### 2.2 Equipment

Langmuir and LB films were prepared using a KSV Nima Langmuir trough (KSV 5000) equipped with a Wilhelmy plate. The pH of the subphase and buffer solutions was measured with a Crison Micro pH 2000 m. The subphase temperature was maintained at 25 °C using a Nelslab thermostat (RTE-111). Infrared spectra were obtained in transmittance mode using a Jasco FT/IR-6600 spectrometer. UV–Vis spectra were acquired with a Shimadzu UV-2600 spectrophotometer. Atomic force microscopy (AFM) images were obtained using a Cypher AFM (Asylum Research, Oxford Instruments). Dilatational surface rheology was performed using the oscillating barrier mode of the Langmuir trough (KSV-Nima). Electrochemical measurements were conducted with a potentiostat/galvanostat (Metrohm Autolab).

### 2.3 Production of Langmuir and LB Films

A chloroform solution containing P3HT and DMPA was prepared at a mass ratio of 0.35:0.65 (w/w) and a concentration of 0.5 mg/mL. ITO substrates were cleaned sequentially with water, soap, and acetone prior to deposition. The mixed solution was spread onto the air–water interface of the Langmuir trough filled with an aqueous solution of KCl 0.1 mol/L. After 15 min to allow solvent evaporation, a laccase solution (30 μL of 0.5 mg/mL) was incorporated to the monolayer as described in refs 27,35 by cospreading the materials. Graphene oxide was dispersed in water to a concentration of 0.5 mg/mL and then 300 μL was incorporated to the monolayer as described in refs 35,37 Films were compressed at a barrier speed of 10 mm/min to the collapse. Transfer onto ITO substrates as Langmuir–Blodgett films was performed after compressing the monolayer to 30 mN m^–1^ and maintaining this surface pressure while the barriers oscillated to ensure equilibration. Once the monolayer stabilized, defined as an uncompensated area variation of less than 1% over 5 min—the solid substrate, previously immersed in the subphase, was withdrawn vertically at 5 mm min^–1^. Multilayer Y-type deposition was achieved by keeping the plate in the emerged position for 10 min to ensure drying before immersion at the same withdrawal speed. No additional waiting time was applied between consecutive immersion and emersion cycles. The transfer efficiency of each layer was evaluated through the transfer ratio, calculated as the ratio between the experimentally transferred area and the theoretical area expected for ideal deposition.

### 2.4 Characterization of the Films

Surface pressure of the floating monolayers was measured by a Wilhelmy plate made of filter paper intercepting orthogonally the air–water interface and followed along the monolayer compression resulting in surface pressure–area isotherms.

In addition, dilatational surface rheology was performed on monolayers compressed to 30 mN/m and stabilized for 10 min. Oscillatory area deformations of 1% were applied at a frequency of 2 mHz for at least 10 cycles, and the average dilatational modulus was calculated.

UV–Vis spectra were recorded between 190 and 1000 nm in absorbance mode using quartz substrates as blanks. AFM images were collected in tapping mode at room temperature using μmash tips (length 160 μm, width 40 μm, thickness 4 μm, spring constant 26 N/m), with a scanning rate of 0.5 Hz.

### 2.5 Electrochemical Measurements

Electrochemical characterization was carried out in a 40 mL three-electrode cell using the LB films deposited on ITO as the working electrode, a platinum plate as the counter electrode, and an Ag/AgCl electrode as reference. The counter electrode was polished, rinsed with Milli-Q water, and flame-annealed before each measurement. Cyclic voltammetry was performed in 0.1 M NaCl with added phenolic analyte, over a potential range of −1.0 to +1.0 V, a scan rate of 0.05 V/s, and 5 cycles. The measurements were repeated at least three times, and variations of less than 5% were obtained and are reported.

## 3 Results and Discussion

### 3.1 Langmuir Films Characterization

To characterize the floating monolayers, surface pressure–area (π–A) isotherms were obtained to investigate the behavior and interactions of the materials incorporated into the pure DMPA monolayer (Figure 1A). DMPA exhibited a characteristic isotherm,^38,39^ with a pseudoplateau marking the transition between liquid-expanded and liquid-condensed phases, followed by a steep rise in surface pressure until collapse above 50 mN/m. Upon addition of the conductive polymer P3HT, the isotherm shifted to higher molecular areas, indicating polymer insertion between the lipid alkyl chains and consequent monolayer expansion. This hybrid monolayer of DMPA and P3HT will hereafter be referred to as the “Mix.” When laccase and graphene oxide were incorporated into the Mix, the isotherm showed signs of condensation, most likely due to attractive interactions between the enzyme and GO in the aqueous subphase and the polar headgroups of the lipid, leading to closer molecular packing within the monolayer.

**Figure 1:**
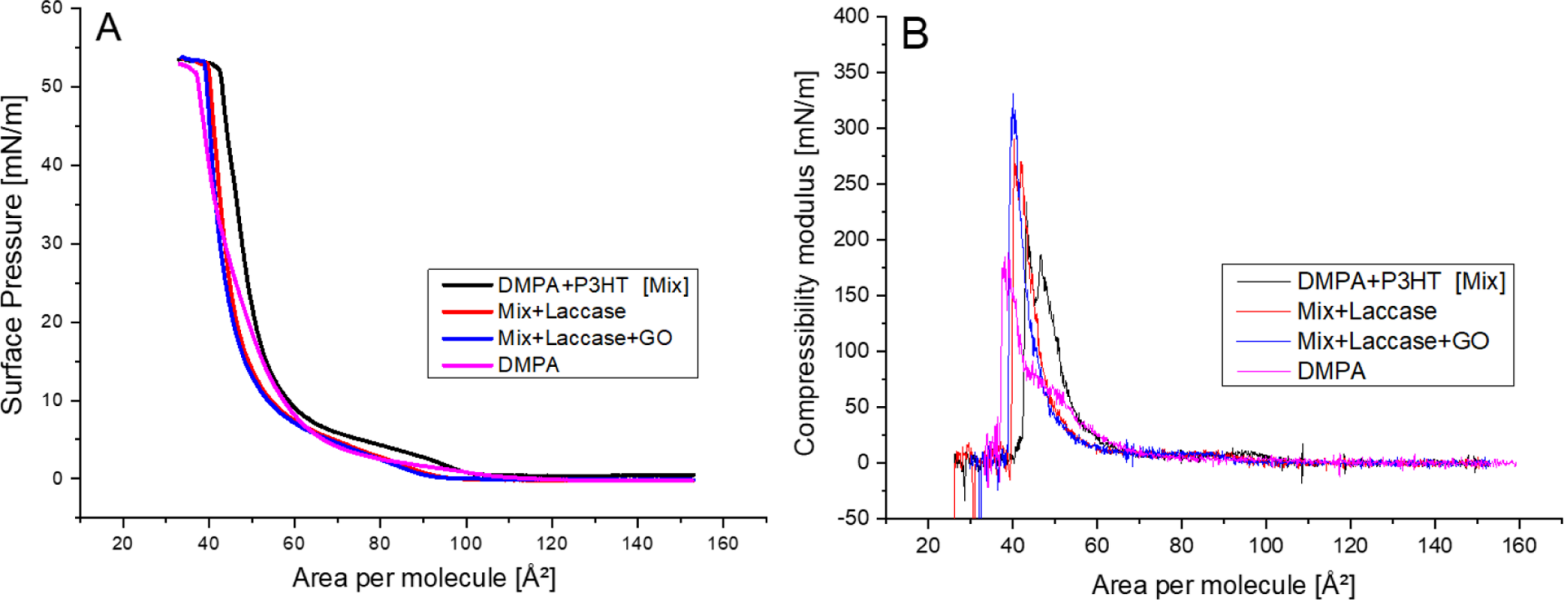
Surface pressure–area (A) and Surface compressibility modulus (B) isotherms for pure DMPA and Mix (blend of DMPA and P3HT) without or with Laccase and GO.

For Langmuir monolayer formation, a KCl subphase was used because potassium ions are known to promote enzyme adsorption at the air–water interface through salting-out effects, a procedure extensively described in the literature for facilitating stable incorporation of proteins into Langmuir and Langmuir–Blodgett films.^35,40–43^ This ionic environment is required only during film preparation and does not reflect the conditions used in electrochemical measurements. In contrast, the electrochemical experiments were performed in NaCl solution, which provides a suitable and inert supporting electrolyte without interfering with the redox behavior of Pyrocatechol or with the activity of the immobilized enzyme. As demonstrated here and in previous work^35^ the laccase-based LB films retain their catalytic activity in pure water and NaCl, indicating that KCl is unnecessary, and indeed undesirable, during voltammetric analysis. Thus, the use of KCl in the subphase and NaCl during electrochemial measurements serves distinct functional purposes appropriate to each technique.

Although GO was incorporated into the system, its presence did not produce a pronounced change in the π–A isotherms. This is expected because GO, added in small amounts and not behaving as a classical amphiphile, interacts mainly with the polar regions of the monolayer rather than contributing significantly to the interfacial area.

It is important to note that a surface pressure–area isotherm for pure P3HT is not presented because P3HT does not form a stable Langmuir monolayer at the air–water interface. As widely reported for conjugated polymers, P3HT exhibits predominantly cohesive rather than adhesive interactions, leading to aggregation and film collapse instead of spreading as an ordered monolayer.^27,44^ Its hydrophobic π-conjugated backbone and strong π–π stacking interactions prevent its stabilization at the interface, precluding the acquisition of a meaningful π–A isotherm. For this reason, P3HT must be cospread with an amphiphilic lipid, here DMPA, which provides interfacial anchoring and enables the formation of a stable mixed monolayer. The increased molecular area observed in the mixed-film isotherm therefore reflects the accommodation of P3HT within the lipid matrix rather than the packing behavior of P3HT alone.

The surface pressure–area (π–A) isotherms were analyzed using the equation proposed by Davies and Rideal, *K* = ${-A\left(\frac{\partial \pi }{\partial A}\right)}_{T}$ ,^45^ to obtain the surface compressibility modulus (*K*), as shown in Figure 1B. This parameter is commonly used to describe the two-dimensional physical states of monolayers, where higher values correspond to increased compressional elasticity. The pure DMPA monolayer exhibited *K* values of up to 175 mN/m, consistent with a liquid-condensed state. Upon successive incorporation of P3HT, laccase, and graphene oxide, the maximum *K* values increased progressively to approximately 250, 300, and 325 mN/m, respectively, accompanied by a leftward shift of the curves, as previously observed in the π–A isotherms. These results indicate that the addition of polar components enhances molecular interactions, reducing lateral repulsion and leading to more compact, rigid monolayers. Consequently, the films withstand compression more sensitively, resulting in a steeper rise in surface pressure at lower molecular areas decrease.

To evaluate the reversibility of the monolayers, the films were subjected to two successive compression–expansion cycles. As extensively reported in the literature, pure DMPA does not exhibit significant hysteresis,^46,47^ as common for saturated phospholipids.^48^ The hysteresis behavior of the other monolayers is shown in Figure 2. For the Mix monolayer (DMPA + P3HT), a small hysteresis was detected between the expansion and subsequent compression cycles, which can be attributed to molecular rearrangements occurring after the initial compression. Upon incorporation of laccase, either in the absence or presence of graphene oxide, both the hysteresis and the extent of rearrangement increased slightly. This behavior suggests stronger intermolecular interactions and a partial restriction of the mobility of the amphiphilic molecules at the interface. As graphene oxide appears to promote additional effect in the monolayer through polar and π–π interactions, it may promote films that are more rigid yet still reversible to a large extent. Overall, these findings demonstrate that the incorporation of P3HT, laccase, and GO modifies the dynamic response of the films, balancing molecular ordering with mechanical reversibility.

**Figure 2:**
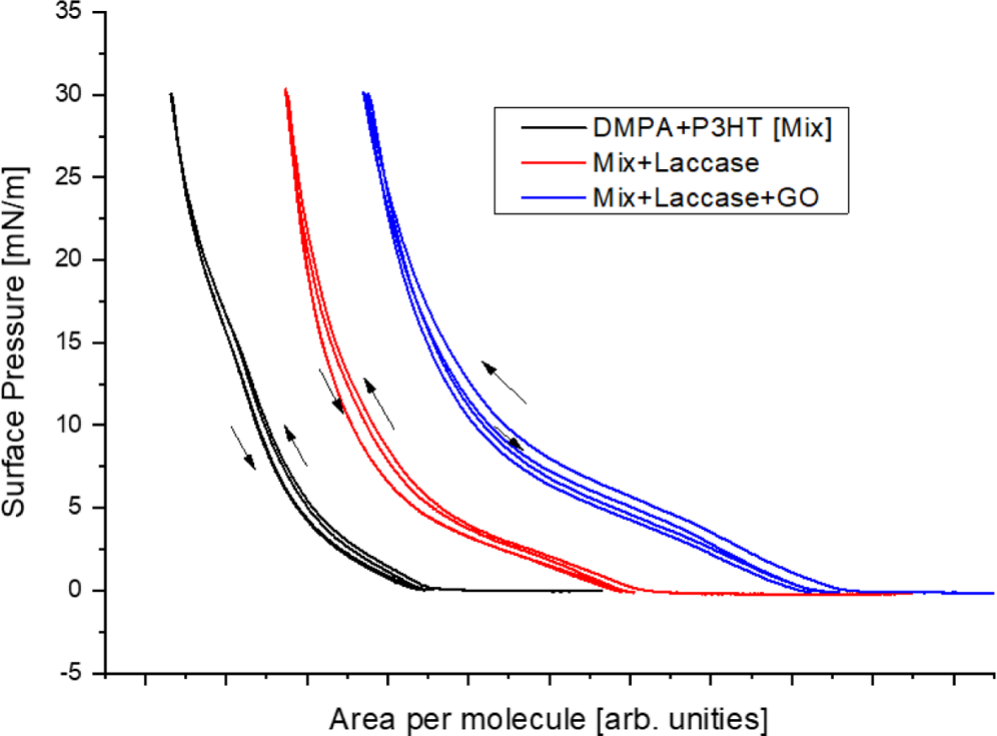
Compression–Expansion surface pressure–area isotherms for Mix monolayers without or with laccase and GO. The curves have been horizontally offset for clearer comparison.

Table 1 shows the dilatational rheology data for monolayers previously compressed to 30 mN/m, followed by barrier oscillations generating short compression–decompression cycles. The dilatational modulus (E*) is a complex parameter composed of an elastic part (*E*′) and a viscous part (*E*″), derived from the relationship between the applied stimulus (area variation) and the system response (surface pressure), along with a phase angle (θ). Higher θ values correspond to a greater viscous contribution. For pure DMPA, previously reported by ref,^35^ E* was 135 mN/m with a phase angle of 0.36 rad, reflecting a predominantly elastic character. When P3HT was introduced, all values revealed a decrease in elasticity, contrasting with the increase in compressional modulus observed from the unidirectional π–A isotherms. This apparent discrepancy arises because the compressional modulus reflects the monolayer’s equilibrium resistance to area reduction, while dilatational rheology probes the dynamic response under oscillatory perturbation. In this case, the incorporation of the polymer increases rigidity at equilibrium but simultaneously introduces dissipative processes (e.g., chain reorganization and interfacial friction), which manifest as reduced elasticity and enhanced viscosity under dynamic conditions. For the Mix monolayer, this effect was particularly evident, with a phase angle of 0.65, indicating a significant viscous contribution.

**Table 1:** Interfacial Dilatational Viscoelastic Properties for Mix Monolayers without or with Laccase (0.5 mg/mL) and GO (0.5 mg/mL)^a^

monolayer	Θ (rad)	E* (mN/m)	*E*’ (mN/m)	*E*” (mN/m)
Mix	0.65	42.76	34.07	25.84
Mix + Lacca	1.06	66.82	32.52	58.38
Mix + Lacca + GO	0.38	126.17	117.15	46.85

a Initial surface pressure = 30 mN/m, frequency= 20 mHz, and 1% of area variation (each data is an average of 10 cycles with error <2%).

With the addition of laccase, E* increased relative to the Mix monolayer, consistent with the enhanced rigidity indicated by the isotherms, while the phase angle also rose, revealing a stronger viscous contribution associated with the enzyme’s bulkier structure and slower relaxation dynamics at the interface. When graphene oxide was incorporated together with laccase, E* increased further, reflecting additional stabilization of the film through π–π and electrostatic interactions, while the phase angle decreased compared to the enzyme-only monolayer. This behavior suggests that GO promotes more efficient packing and reduces molecular rearrangements, thereby lowering energy dissipation. In summary, the dilatational rheology results corroborate the isotherm analysis, demonstrating that while P3HT introduces viscosity and reduces elasticity, the combination of laccase and GO leads to stiffer, more ordered, and dynamically stable monolayers.

### 3.2 LB Films Characterization

The transfer ratios during LB deposition (Table S1 Supporting Information) showed efficient and stable transfer for the first three layers, with values close to unity. Beyond this point, saturation occurred, and thicker films could not be formed, as indicated by negative ratios on the downstroke and positive ratios on the upstroke, evidencing instability in the transfer process.

Figure 3 shows the evolution of UV–Vis absorption for each odd layer of the Mix + Laccase films, with or without GO, corresponding to the upward strokes during LB deposition. *T. versicolor* laccase exhibits a characteristic absorption near 245 nm,^49^ while P3HT presents a band around 490 nm in solution.^50^ The were obtained from a wider spectrum (Supporting Information), which were consistent with these features; however, overlap between the polymer and enzyme absorption regions prevents confirmation of laccase incorporation by this method.^49^ Despite the material loss observed after the third layer, the polymer signal increased with each deposition cycle for the films without GO. For Mix + Laccase + GO films, a more irregular trend was observed for the P3HT band intensity, likely due to irregular transfer behavior during negative ratio strokes, suggesting that stable films beyond three layers are not achievable.

**Figure 3:**
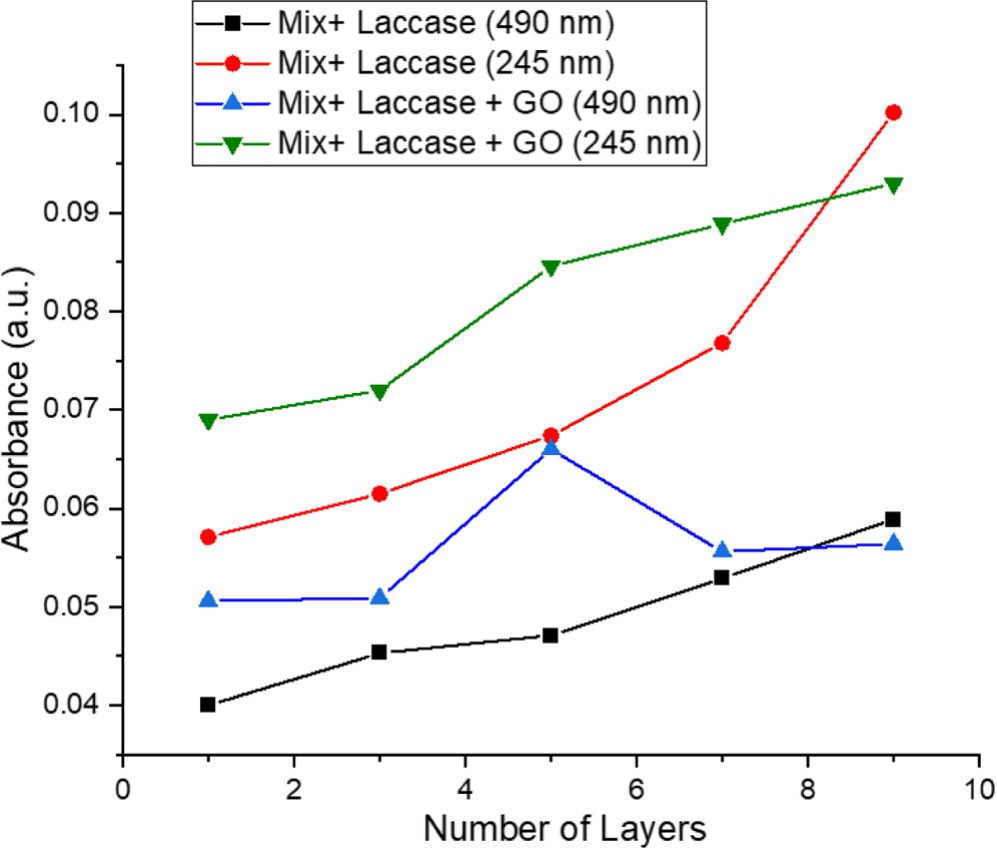
Evolution of the absorbance of Langmuir–Blodgett (LB) films at selected wavelengths. The connecting lines are provided solely as visual guides.

The increase in UV–Vis absorbance with each deposited Langmuir–Blodgett layer does not follow a perfectly linear trend because the transfer of material onto the solid substrate is not strictly uniform throughout the multilayer assembly. As shown in the Supporting Information, the transfer ratios become unstable after the third layer, meaning that the amount of material effectively transferred during each upward stroke varies. This leads to cycles in which slightly more or slightly less material is deposited, producing the small deviations from ideal linearity in the absorbance versus number of layers plot. Additionally, the mixed composition of the films (DMPA, P3HT, laccase, and in some cases GO) contributes to nonhomogeneous packing at the interface and during deposition. Macromolecules such as laccase, and particularly the presence of graphene oxide sheets, may reorganize or partially desorb during transfer, further contributing to the irregular increment in absorbance. However, from a practical point of view, the first three layers, where the transfer ratio is close to unity, show increments in absorbance that are approximately linear. In this region, deposition is more reproducible and the LB transfer is efficient, so the absorbance increases proportionally to the number of layers. Thus, depending on the analytical perspective, one might reasonably consider that portions of the curves exhibit linear behavior, even though the complete set of points does not comply with perfect linearity.

Similar nonlinear dependences of UV–Vis absorbance on the number of LB layers have been reported in the literature. For asymmetric bent-core liquid crystal LB films, the absorbance initially increases but then decreases beyond 5–10 layers, evidencing irregular multilayer growth and partial collapse of the stack.^51^ In DNA-containing LB films, the absorbance ceases to grow linearly after about ten layers, indicating less material deposition per cycle as surface roughness increases and electrostatic interactions weaken.^52^ For polymer/carbon nanotube LB films, the absorbance shows a pronounced saturation at high layer numbers, with negligible changes in optical density despite additional layers.^53^ These examples support the view that deviations from linear absorbance–thickness behavior are common in multilayer LB architectures when transfer ratios decrease, roughness increases, or aggregation and collapse processes become relevant.

AFM images in Figure 4 show the morphology of single-layer films with different compositions, highlighting distinct roughness values. The film containing only DMPA and P3HT exhibited moderate roughness (*R*q = 1.528 nm). As expected, the incorporation of laccase increased surface roughness due to the macromolecular size of the enzyme (*R*q = 2.150 nm). Interestingly, the addition of graphene oxide led to a marked decrease in roughness (*R*q = 0.835 nm), likely because GO sheets accommodated at the interface, promoting a more homogeneous and compact surface.

**Figure 4:**
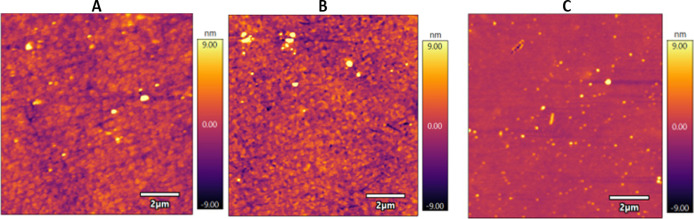
AFM images for 1-layer LB films. (A) Mix. (B) Mix + Laccase. (C) Mix + Laccase + GO.

The AFM results obtained for the different film compositions are consistent with trends reported in the literature for lipid–polymer–enzyme and graphene oxide–based Langmuir–Blodgett assemblies. The increase in roughness observed when laccase is incorporated into the DMPA/P3HT matrix reflects the typical morphological effect produced by immobilizing bulky biomacromolecules at interfaces. Enzymes usually induce nanoscale heterogeneity and height fluctuations because their globular domains partially protrude from the film surface, as previously described for oxidase-containing LB films and other protein–lipid architectures.^54,55^ In contrast, the marked reduction in roughness upon incorporation of graphene oxide correlates well with studies demonstrating that GO sheets act as planar, rigid fillers that occupy interfacial voids and promote more homogeneous packing^26,37^ Owing to their extended 2D geometry and strong interactions with both lipids and polymers, GO nanosheets tend to flatten the film surface and decrease height variations, producing smoother, more compact morphologies. This behavior is fully consistent with the morphology observed here for the Mix + Laccase + GO film, confirming that GO contributes to structural homogenization and improved surface organization in the hybrid LB assembly.

Although the π–A isotherms of the Mix + Laccase and Mix + Laccase + GO films do not show pronounced macroscopic differences, several complementary measurements demonstrate that GO promotes a more compact interfacial structure. GO does not behave as a classical amphiphile and therefore contributes minimally to the molecular area recorded in the isotherm; its effects instead manifest at the microstructural level. In the presence of GO, the compressibility modulus increases, dilatational rheology reveals higher elasticity with reduced molecular rearrangement, and AFM images show a marked decrease in surface roughness with a more homogeneous morphology. Together, these results indicate that GO fills interfacial voids and enhances molecular packing, leading to a more compact and organized film even though this refinement is not strongly reflected in the surface pressure–area isotherms.

### 3.3 Testing LB Film as Working Electrode by Cyclic Voltammetry

It is important to emphasize that all electrochemical experiments were performed using single-layer LB films. This choice is intentional: multilayer architectures containing entrapped enzymes are well-known to cause diffusion limitations and hinder substrate accessibility, a phenomenon extensively reported in the literature not only for laccase but for a wide range of immobilized enzymes.^56–60^ Using one-layer films ensures optimal diffusion, preserves enzymatic activity, and provides more reliable electrochemical responses.

Cyclic voltammetry was performed in a three-electrode cell using ITO coated with the films as the working electrode (active area 1 cm^2^), a platinum plate as the counter electrode, and an Ag/AgCl (3 M) electrode as reference. A 0.1 mol/L NaCl solution in Milli-Q water served as the supporting electrolyte and was also used for blank measurements during LOD (limit of detection) and LOQ (limit of quantification) tests.

In preliminary tests, we evaluated the use of citrate and phosphate-based buffers to control the pH during Pyrocatechol detection. However, citrate buffer completely suppressed the electrochemical response of the Pyrocatechol oxidation product, preventing reliable signal acquisition, and PBS produced a similar attenuation of the redox peaks. For this reason, buffer solutions were not suitable for this system. All measurements were therefore carried out in pure water, a condition that does not impair the enzymatic activity of the immobilized laccase. This behavior is consistent with our previous findings,^26^ where laccase incorporated into DMPA/P3HT/GO Langmuir–Blodgett films exhibited stable catalytic performance and well-defined voltammetric responses in pure water. The robust microenvironment provided by the LB architecture preserves the enzyme’s activity without requiring external buffering, and attempts to adjust pH through buffer addition resulted in signal suppression rather than improvement. Consequently, pure water was selected as the optimal medium for maintaining enzyme function while ensuring a measurable and reproducible analytical signal.

The voltammograms in Figure 5 show that covering bare ITO with LB films decreased the overall signal, but enzyme immobilization conferred selectivity toward phenolics, here exemplified by Pyrocatechol. With the incorporation of graphene oxide, the anodic and cathodic peak intensities increased markedly, reflecting its role in enhancing electron transfer and facilitating redox processes.

**Figure 5:**
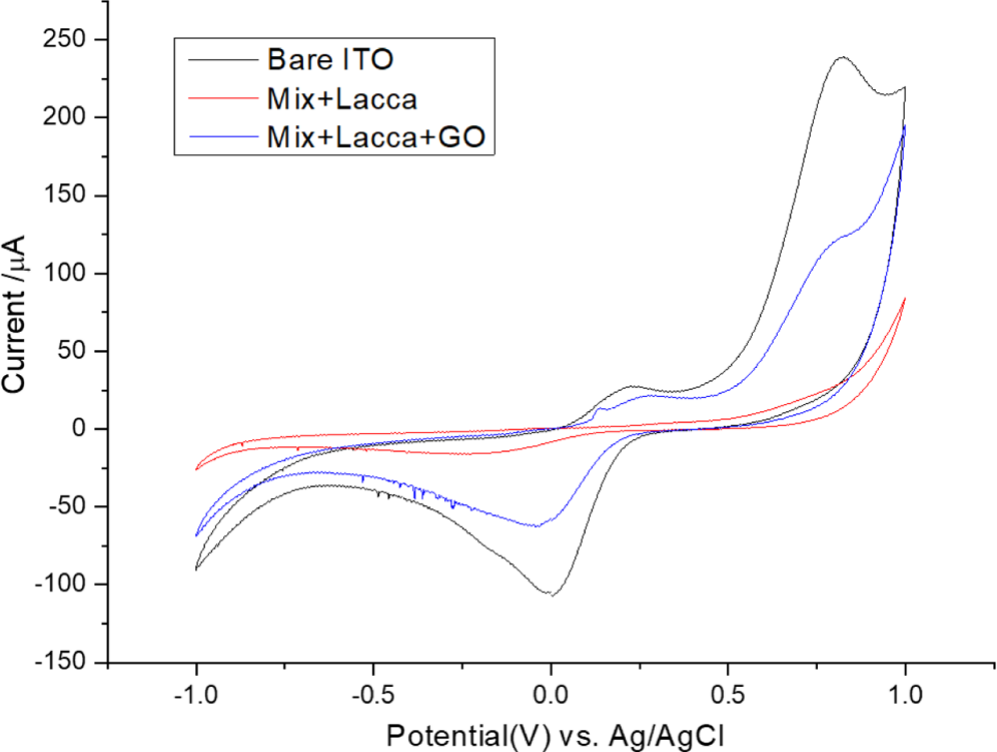
Voltammograms comparing signal intensity with bare ITO, 1-layer Mix + Laccase and 1-layer Mix + Laccase + GO, Pyrocatechol 1 × 10^–3^ mol/L.

Although the voltammograms exhibit low-intensity signals inherent to ultrathin monolayer LB films, stability and layer-fixation tests confirmed that the films remain structurally robust during electrochemical cycling. Repeated potential scans showed that the voltammetric profile and peak positions were preserved, with variations in peak current remaining minor after successive cycles, indicating minimal loss of material or enzymatic activity. These results demonstrate that the Langmuir–Blodgett films are stably anchored to the ITO substrate and maintain their integrity under the applied electrochemical conditions.

#### 3.3.1 Evaluation of Sensors against Pyrocatechol

Figure 6 shows the cyclic voltammograms obtained with a working electrode containing a single layer of Mix + Laccase, as the Pyrocatechol concentration was varied from 10^–7^ to 10^–3^ mol·L^–1^. The cathodic peak, attributed to the reduction of Pyrocatechol quinone, was selected as the analytical signal for calibration. From this, the calibration curve shown in Figure 7B was constructed, displaying a sensitivity of 29,638 μA·mol^–1^·L and an excellent linear correlation (*R* ^2^ = 0.989). The linear dynamic range extended from 1.64 × 10^–6^ to 8.29 × 10^–4^ mol·L^–1^, demonstrating the sensor’s ability to reliably quantify Pyrocatechol across several orders of magnitude.

**Figure 6:**
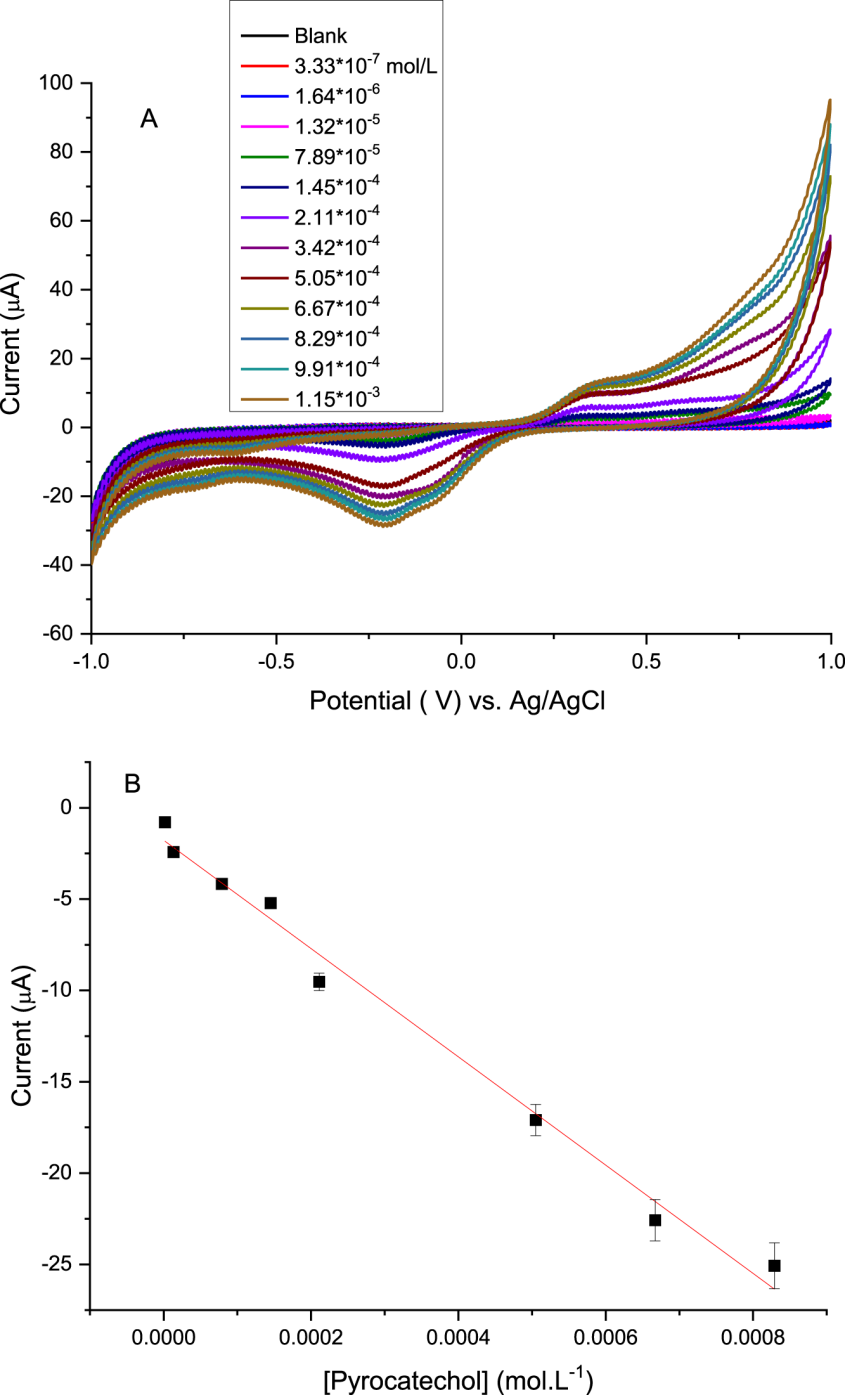
(A) Cyclic voltammogram varying the Pyrocatechol concentration from 3.33 × 10^–7^ to 1.15.10 ^–3^ molL^–1^; (B) Calibration curve, Pyrocatechol concentration *x* cathodic peak.1-layer Mix + Laccase LB film was used as working electrode. The calibration curves were obtained from three independent measurements using separately prepared electrodes separated in the linear region. The relative deviation for each data point was below 5%.

**Figure 7:**
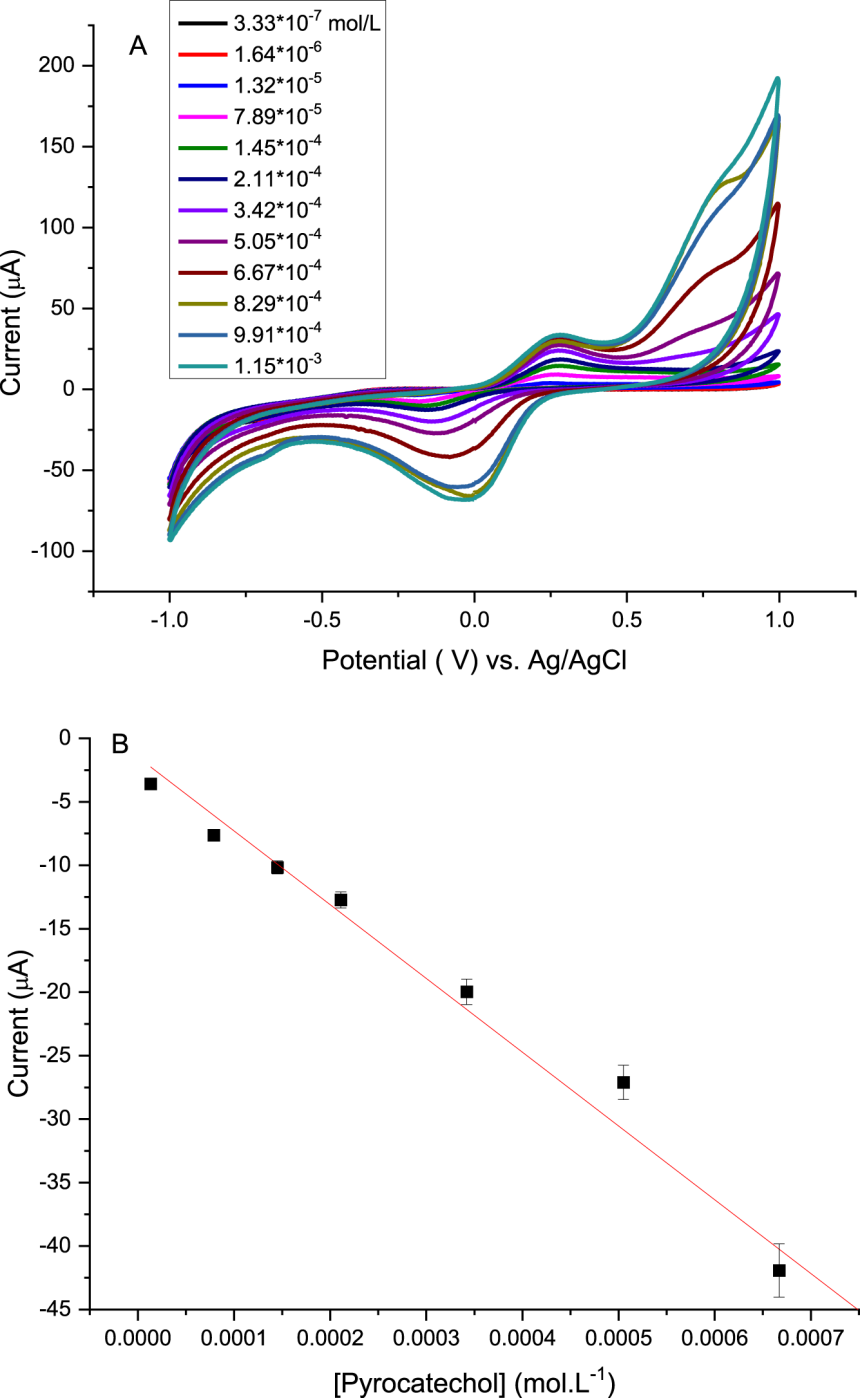
Using 1-layer Mix + Laccase + Graphene oxide LB film as working electrode; (A) cyclic voltammogram varying the Pyrocatechol concentration from 3.33 × 10^–7^ to 1.15 × 10 ^–3^ molL^–1^; (B) calibration curve, Pyrocatechol concentration *x* cathodic peak. The calibration curves were obtained from three independent measurements using separately prepared electrodes separated in the linear region. The relative deviation for each data point was below 5%.

Figure 7 presents the cyclic voltammograms obtained for the sensor with one layer of Mix + Laccase + Graphene Oxide over the same concentration range as before. The cathodic peak was again used to construct the calibration curve (Figure 7B). Incorporation of graphene oxide led to a marked improvement in performance, with the sensitivity increasing to 58,118 μA·mol^–1^·L, a stronger linear correlation (*R* ^2^ = 0.993), and an extended linear range from 1.32 × 10^–5^ to 1.15 × 10^–3^ mol·L^–1^. Compared with the Mix + Laccase film, the presence of graphene oxide nearly doubled sensitivity while also broadening the quantifiable concentration window, underscoring its key role in enhancing sensor efficiency.

A slight nonlinear behavior is observed in the Pyrocatechol detection data at higher concentrations (Figure 7). This deviation from ideal linearity is expected for enzyme-modified ultrathin films, where the current response reflects not only diffusion but also adsorption phenomena, partial enzyme–substrate interactions, and interfacial electron-transfer limitations. Such mixed kinetic regimes have been widely reported for laccase-based and other enzymatic sensors using LB films. Importantly, the concentration range used for analytical quantification displays excellent linear correlation, and the nonlinearity appears only near the upper limit, where saturation effects and increased electron-transfer resistance can occur. This behavior is therefore consistent with the electrochemical characteristics of Pyrocatechol in organized enzymatic interfaces.^27,57,58^

The LOD and LOQ were calculated for both sensors, with and without graphene oxide, and are summarized in Table 2 along with sensitivity and *R* ^2^ values for direct comparison. Incorporation of graphene oxide significantly enhanced performance, lowering the LOD by nearly 1 order of magnitude and reducing the LOQ accordingly. This improvement can be attributed to the facilitation of electron transfer between the analyte and the electrode surface—an essential factor in redox processes. Furthermore, the cathodic peak shifted toward potentials closer to zero, showing that less energy is required for analyte detection when graphene is present, thereby improving both sensitivity and efficiency of the sensor.

**Table 2:** Comparison between Mix + Laccase Sensors with and without Graphene Oxide with Values of Detection and Quantification Limits, Peak Catodic Potencial with 1.15 mol L^–1^ Pyrocatechol, Linear Correlation and Sensibility

sensor	*E*pc [1.15 × 10^–3^ mol L^–1^]	LOD	LOQ	*R* ^2^	sensibility
1-layer Mix + Laccase	–0.2	3.39 × 10^–6^	1.13 × 10^–5^	0.989	–29638
1-layer Mix + Laccase + GO	0	2.08 × 10^–6^	6.95 × 10^–6^	0.994	–58118

The limits of detection (LOD) and quantification (LOQ) were calculated using standard analytical expressions based on the standard deviation of the blank and the slope of the calibration curve;

$$LOD=\frac{3\sigma }{S}$$

$LOQ=\frac{10\sigma }{S}$ where σ is the standard deviation of the blank current and *S* is the sensitivity (slope of the calibration plot). Using these equations, the Mix + Laccase film exhibited an LOD of 3.39 × 10^–6^ mol L^–1^ and an LOQ of 1.13 × 10^–5^ mol L^–1^, whereas incorporation of graphene oxide significantly improved analytical performance, lowering the LOD to 2.08 × 10^–6^ mol L^–1^ and the LOQ to 6.95 × 10^–6^ mol L^–1^. These results demonstrate that GO enhances electron transfer and increases the effective electroactive area, leading to higher peak currents and therefore better detection limits.

Comparison with the literature further highlights the improved performance of the GO-containing LB films. Cabaj et al.^54^ reported LOD values between 10^–5^and 10^–6^ mol L^–1^for laccase immobilized in conjugated–polymer LB films for phenolic analytes, which is comparable to the non-GO film here but less sensitive than our GO-containing sensor. Bragazzi et al. described laccase films with detection limits around 10^–6^ mol L^–1^for pharmaceutical analytes,^55^ again consistent with our results but without enhancement by carbon nanomaterials. More recently, Scholl et al. demonstrated that graphene oxide integrated into lipid LB films significantly enhances electron transfer and reduces overpotentials in enzymatic sensors, though they did not directly report LOD values.^37^ Our findings align with this behavior, combining GO’s interfacial organization with laccase’s catalytic activity to improve sensor response.

Comparisons with graphene-based nanozyme or hybrid electrodes also confirm the beneficial role of GO. Şenocak and Yıldız reported an ultralow LOD of 3.3 × 10^–10^ mol L^–1^ for Pyrocatechol using Au-decorated GO nanozymes,^56^ but these systems rely on catalytic nanoparticles rather than LB-organized enzyme films and are therefore not directly comparable. When compared to LB-based biosensors in the literature, the present GO-incorporated films show equal or superior sensitivity within the typical micromolar detection range, while retaining the structural organization and biomimetic advantages of Langmuir–Blodgett architectures.

Overall, incorporating GO nearly doubled the sensitivity and reduced LOD values in agreement with previous studies on carbon-nanomaterial-enhanced LB films, confirming that GO plays a key role in improving electron transfer and lowering the energy barrier for analyte detection.

Also, we emphasize that the lower *R* ^2^ values and the slight nonlinear behavior observed in Figures 8 and 9 arise from the quasi-reversible nature of Pyrocatechol redox processes on enzyme-modified LB films. In such systems, deviations from ideal linearity are expected because the peak current is influenced not only by diffusion but also by adsorption, partial enzyme–substrate interaction, and interfacial reorganization during redox cycling. Similar nonideal or nonlinear scan-rate dependences have been widely reported for laccase-based electrodes and other enzymatic or nanostructured interfaces, where mixed kinetic regimes lead to curvature or decreased *R* ^2^ in Randles–Ševčík plot.^55,56^ Moreover, graphene- or polymer-containing LB films frequently display heterogeneous electron-transfer domains, producing modest deviations from linearity as also noted by Scholl et al.^37^

**Figure 8:**
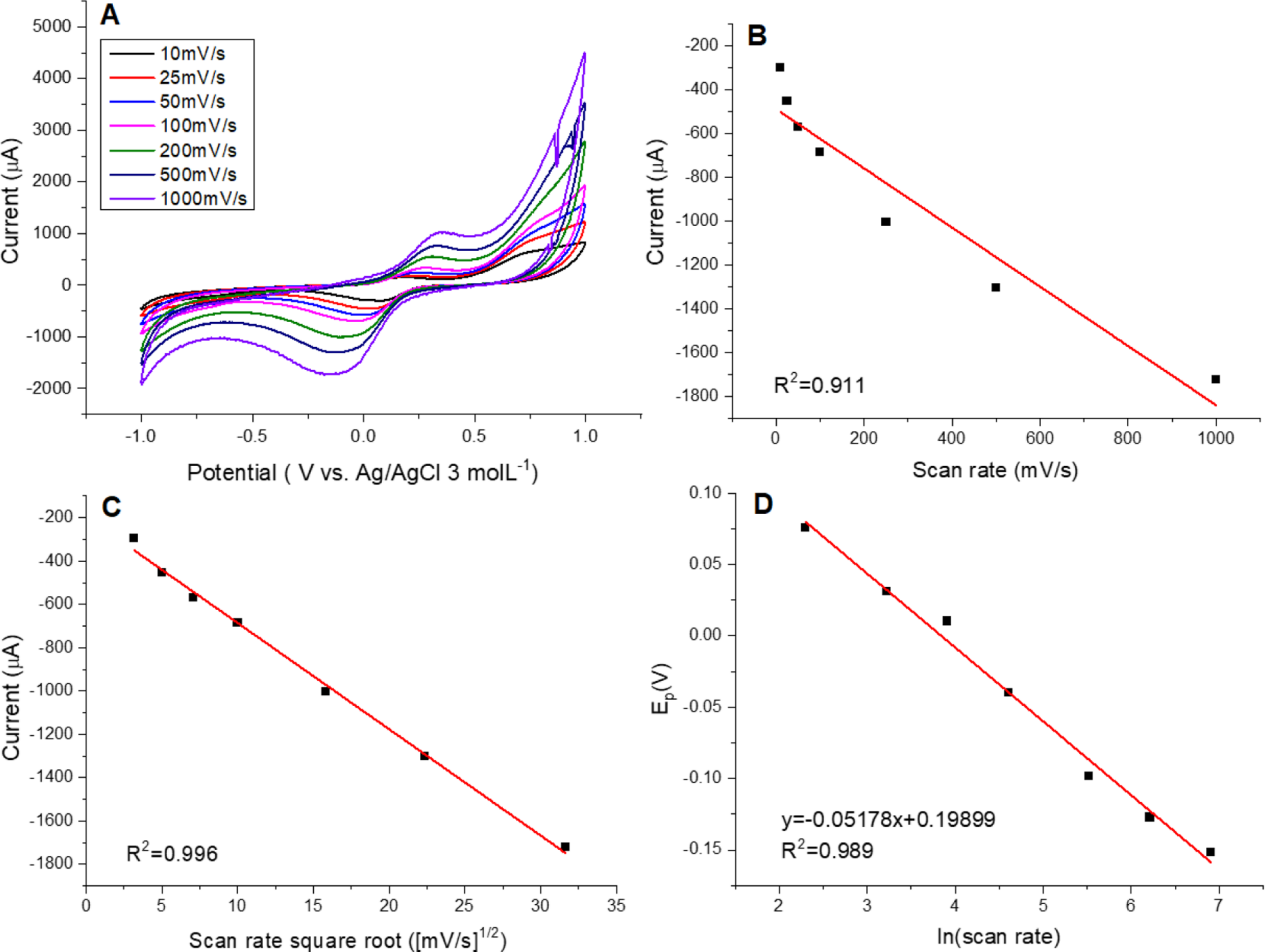
Electrochemical behavior of Pyrocatechol using a Mix + Laccase LB film as the working electrode. (A) Cyclic voltammograms recorded at varying scan rates with Pyrocatechol concentration fixed at 1.15 × 10^–3^ mol L^–1^. (B) Plot of cathodic peak current versus scan rate. (C) Plot of cathodic peak current versus the square root of the scan rate. (D) Plot of cathodic peak potential versus the natural logarithm of the scan rate.

**Figure 9:**
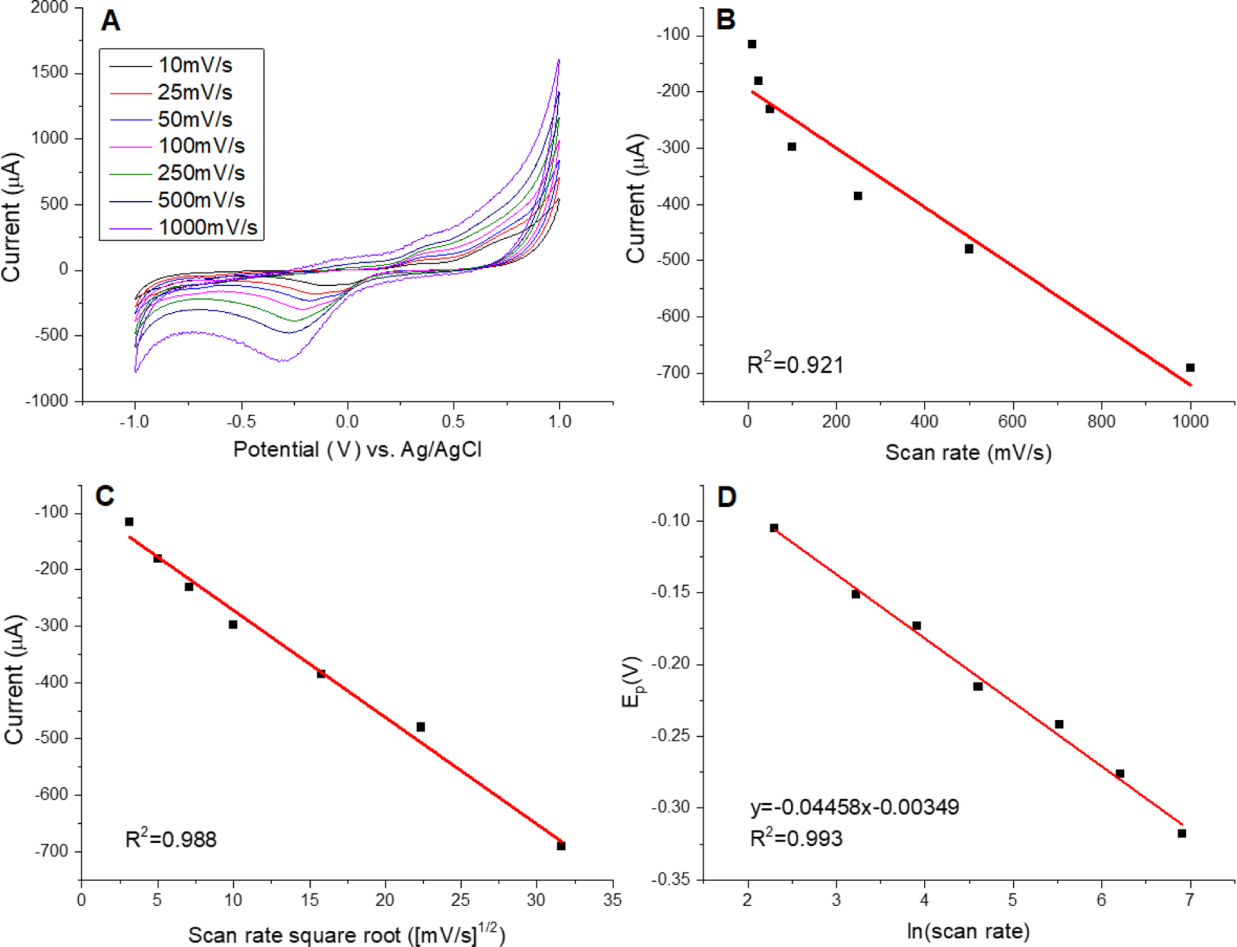
Electrochemical response of Pyrocatechol using a Mix + Laccase + Graphene Oxide LB film as the working electrode. (A) Cyclic voltammograms recorded at varying scan rates with Pyrocatechol concentration fixed at 1.15 × 10^–3^ mol L^–1^. (B) Cathodic peak current as a function of scan rate. (C) Cathodic peak current as a function of the square root of the scan rate. (D) Cathodic peak potential as a function of the natural logarithm of the scan rate.

In Supporting Information (Figure S2), we show the cyclic voltammograms of sensors constructed with nine layers of Mix + Laccase without graphene (A) and with graphene (B) in response to Pyrocatechol. For the film without graphene, no well-defined peaks were observed; only an increase in background current with rising Pyrocatechol concentration was detected, likely due to the accumulation of ionic species in the medium. This indicates that the multilayer sensor lacks specificity and is unsuitable for Pyrocatechol detection. In the case of the nine-layer graphene-containing film, peaks corresponding to Pyrocatechol oxidation and quinone reduction were present, but the currents were highly suppressed and the peak shapes were distorted compared to the single-layer sensor. These results demonstrate that multilayer films are unstable and ineffective, consistent with the negative transfer ratios observed during deposition, and therefore the construction of sensors beyond three layers was discarded.

Lipid-based Langmuir–Blodgett films are known to exhibit limited and often unstable multilayer growth, typically restricted to only a few layers. This behavior was also observed in our system, where the transfer ratios decreased markedly after the third deposition, indicating that true multilayer buildup was not achieved. The nominal 9-layer films were therefore included only as a control to demonstrate that successive depositions do not produce functional thickening: the resulting films displayed suppressed electrochemical responses and poorly defined peaks, confirming the absence of effective layer growth. In contrast, single-layer films provided the best performance, as they avoid the diffusion limitations and enzyme entrapment commonly reported for multilayer LB assemblies. For these reasons, all analytical measurements were carried out using 1-layer sensors, while the 9-layer experiments served to reinforce the intrinsic growth limitations of this type of film.

Also, the rationale for comparing 1-layer and nominal 9-layer films is to contrast the thinnest and thickest configurations obtainable in our system, rather than to evaluate incremental growth between intermediate layers. This approach is consistent with the literature, which shows that single-layer LB films typically provide optimal enzymatic activity, while inner layers of immobilized enzymes often become diffusion-limited and largely inactive. In our case, although true multilayer growth does not occur beyond the first few depositions, the nominal 9-layer film serves as a useful control to demonstrate the loss of electrochemical performance when the system is forced into a thicker configuration. This comparison highlights both the intrinsic growth limitations of lipid-based LB films and the functional superiority of the single-layer architecture for biosensing applications.^56–60^

According to the Randles–Ševčík eq (eq 1), the peak current is directly proportional to the square root of the scan rate for diffusion-controlled processes, whereas linearity with the scan rate indicates adsorption control. The higher Pyrocatechol concentration used in the fixed-concentration voltammetric evaluation was chosen solely to obtain a well-defined redox signal for comparing the electrochemical behavior of the different LB-film configurations. At low concentrations, the currents generated by single-layer LB films are inherently small, which makes it difficult to distinguish differences in peak shape, potential, and electron-transfer efficiency between Mix + Laccase and Mix + Laccase + GO films. Using a higher concentration therefore allows clearer visualization of these comparative effects. It is important to note that all analytical parameters (LOD, LOQ, linearity, and sensitivity) were determined exclusively in the low-concentration range, where Pyrocatechol remains in monomeric form and no additional peaks associated with intermolecular coupling are observed. The higher concentration was thus used only for mechanistic comparison, not for analytical quantification.

Figure 8A shows the cyclic voltammograms of the Mix + Laccase sensor recorded at different scan rates. As expected for Pyrocatechol, whose redox reaction is known to be quasi-reversible, the peak current varied linearly with the square root of the scan rate (Figure 8B,C), confirming that the process is diffusion-controlled.

1 $$i_{p}=2,69\times 10^{5}\cdot n^{3/2}\cdot A\cdot C\cdot \sqrt{D\cdot \nu }$$

According to Laviron’s eq (eq 2) for quasi-reversible systems, the cathodic peak can be used to calculate the charge transfer coefficient (α) and the heterogeneous charge transfer rate constant (*k*_0_).^61^ From the linear fit in Figure 8D, the slope (S) and intercept (b) were obtained and applied to eqs 4 and 5. The α value for this sensor was 0.71, indicating an irreversible system with asymmetric activation energy barriers, where reduction occurs with a lower barrier than oxidation. The calculated *k*_0_ was 2.3 × 10^2^ cm/s, far above the expected range of 10^–2^ to 10^–4^ cm/s. This anomalously high value is likely due to the very low intercept, which artificially inflated the result. Nonetheless, the outcome suggests that the presence of laccase, P3HT, and the nanostructured LB architecture favors charge transfer at the interface. Because the absolute *k*_0_ is unrealistic, it is considered here only as a reference for comparison with the graphene-containing sensor.

2 $$E_{p}=E^{0}-\frac{RT}{\left(1-\propto \right)nF}\ln\left(\frac{RTk^{0}}{\left(1-\propto \right)nF}\right)-\frac{RT}{\left(1-\propto \right)nF}\ln v$$

3 $$S=-\frac{RT}{\left(1-\propto \right)nF}$$

4 $$\propto =1+\frac{RT}{nFS}$$

5 $$k^{0}=\frac{\propto nF}{RT}e^{\left(b-E^{0}/S\right)}$$

It is important to mention that in Figure 8B, the plots appear to exhibit two distinct linear regions with different slopes. This behavior is commonly observed in Langmuir–Blodgett (LB) film–based electrodes,^26^ where different scan rate ranges often correspond to distinct charge-transfer mechanisms. At lower scan rates, the process is typically controlled by diffusion or interfacial adsorption, while at higher scan rates, it may become limited by surface kinetics or capacitive effects, leading to the observed change in slope.

Figure 9 shows the cyclic voltammograms of the graphene oxide–containing sensor at different scan rates. As observed in Figure 9B,C, the peak current varied linearly with the square root of the scan rate, confirming that the redox process remains diffusion-controlled, independent of the presence of graphene. From Laviron’s equation, the kinetic parameters α and *k*_0_ were determined as 0.98 and 1.6 × 10^–3^ cm/s, respectively. The incorporation of graphene oxide increased the asymmetry between the activation energy barriers of the forward and reverse reactions, with the reverse pathway exhibiting a lower barrier. In contrast to the unrealistically high *k*_0_ value obtained for the nongraphene sensor, the result here falls within the expected range (10^–2^–10^–4^ cm/s), supporting its reliability. However, the lower *k*_0_ also indicates that charge transfer is slower and less facilitated when graphene oxide is present, despite its beneficial effects on sensitivity.

The comparison between the results obtained in similar studies from the literature^27,35^ highlights important advances in the electrochemical performance of Langmuir–Blodgett film-based biosensors. In the first study,^21^ using ODA/P3HT as a matrix for laccase immobilization, the sensors exhibited good sensitivity and detection limits on the order of 10^–6^ to 10^–7^ mol·L^–1^ for different phenols (vanillin, Pyrocatechol, and pyrogallol). However, the response showed no correlation with film thickness, confirming that the electrochemical reaction occurs predominantly at the surface. In the present study, by employing DMPA as the lipid component and incorporating graphene oxide, a substantially higher sensitivity was achieved: for Pyrocatechol, the GO-containing sensor reached 5.8 × 10^4^ μA·mol^–1^·L with excellent linearity (*R* ^2^ = 0.993), along with an extended linear range compared to the system without graphene. These improvements are reflected in lower detection and quantification limits, indicating that GO not only stabilizes the film and reduces roughness but also facilitates electron transfer between the enzyme and the electrode. A comparison with Marinho et al.,^35^ who reportedDMPA + LAC + GO films applied to both biosensors and biosupercapacitors, shows that the presence of GO likewise enhanced catalytic activity and improved voltammetric response, although in that study the emphasis was more on multifunctionality (charge storage and detection) than on analytical sensitivity. Taken together, the three studies demonstrate how the choice of lipid matrix, the use of conductive polymers, and the incorporation of nanomaterials such as GO decisively modulate the electroanalytical response and applicability of LB films for phenol detection.

Finally, we emphasize that the reproducibility of the sensor was assessed using at least three independently prepared LB-film electrodes under identical conditions. The cathodic peak currents exhibited relative standard deviations within the 3–10% range, which, together with the short-term operational stability and satisfactory storage stability observed, are consistent with values reported in the literature and are widely accepted as reliable indicators of sensor robustness.^27,37,54^

Also important to report that, in general, a range of studies illustrate how Langmuir–Blodgett and other ultrathin films can be engineered to enhance laccase-based biosensing through different strategies. Our GO-containing DMPA/P3HT/laccase films nearly doubled sensitivity compared to the non-GO system (from 29,638 to 58,118 μA·mol^–1^·L^–1^) and lowered the detection limit to 2.08 × 10^–6^ M, with graphene oxide promoting film condensation, reduced roughness, and improved electron transfer at micromolar concentrations. In contrast, Au-rGO/screen-printed electrodes function as nanozymes, achieving sensitivities of 48,000 μA·mol^–1^·L^–1^ and ultralow detection limits down to 3.3 × 10^–10^ M by exploiting reduced graphene oxide with gold nanoparticles to mimic laccase activity.^62^ These approaches demonstrate complementary routes: enzyme-assisted sensing with high sensitivity in environmentally relevant ranges and enzyme-free nanozyme platforms optimized for trace-level detection.

Other LB-based systems emphasize stability. Medina-Plaza et al.^63^ developed a “bioelectronic tongue” combining lipid matrices, oxidases, and lutetium bisphthalocyanine to discriminate phenolic mixtures with detection limits as low as 10^–8^ M, showing the potential of LB architectures for multicomponent analysis. Bragazzi et al.^55^ employed recombinant fungal laccase films to detect clomipramine with high sensitivity (440 nA·μM^–1^) and rapid responses, underscoring the adaptability of LB films for therapeutic monitoring. Cabaj and co-workers^55,64–66^ demonstrated how conjugated organic mediators, such as diphenylamine derivatives or carbazole-based amphiphiles, enhance electron transfer in laccase or tyrosinase films, increasing catalytic activity up to 3-fold and extending operational stability across multiple cycles. Compared to our GO-based films, which leverage nanocarbon conductivity to maximize sensitivity and lower detection limits, these mediator-assisted systems highlight complementary strategies centered on stability and long-term usability.

The electrochemical processes observed in the voltammograms correspond to the well-known quasi-reversible redox couple of Pyrocatechol and *o*-quinone. The anodic peak arises from the two-electron oxidation of Pyrocatechol to *o*-quinone, while the cathodic peak reflects the subsequent reduction of *o*-quinone back to Pyrocatechol. In the presence of laccase, the anodic response is amplified due to the enzymatic oxidation pathway, which facilitates electron transfer and enhances the generation of the quinone species. Incorporation of GO further increases the peak currents and decreases the overpotential, consistent with its ability to improve interfacial conductivity and promote faster electron-transfer kinetics.^65–67^ These assignments and mechanistic considerations provide a clear interpretation of the redox processes involved and support the observed enhancements in electrochemical performance.

Collectively, these studies reveal the versatility of LB platforms, where conductive nanocarbons and conjugated mediators represent parallel and synergistic tools to optimize biosensor performance across diverse analytical contexts.

## 4 Conclusions

The immobilization of laccase and graphene oxide within a DMPA/P3HT matrix using the Langmuir–Blodgett (LB) technique was successfully achieved. In the Langmuir monolayers, both laccase and graphene oxide promoted film condensation with minimal hysteresis, evidencing stable molecular organization. UV–Vis confirmed effective transfer to solid substrates, while AFM revealed that graphene oxide reduced surface roughness, likely by occupying interfacial regions not filled by the enzyme and promoting a more compact morphology.

Electrochemical characterization using Pyrocatechol as a model analyte demonstrated that graphene oxide substantially enhanced biosensor performance. Its incorporation increased sensitivity, lowered the limits of detection and quantification, amplified the analytical signal, and shifted the reduction potential toward zero, reducing the energy required for detection. Although graphene oxide introduced greater asymmetry between activation energy barriers and slightly slowed charge transfer, these effects did not compromise the overall electroanalytical response.

Together, these results highlight the potential of nanostructured DMPA/P3HT LB films incorporating laccase and graphene oxide as sustainable, high-performance biosensing platforms. Their tunable structure and electrochemical efficiency make them especially promising for future applications in environmental monitoring and food quality control.

## Supplementary Material



## Notes

Supporting Information provides the transfer ratios of the LB films (Table S1), UV–vis spectra (Figure S1), and cyclic voltammograms recorded at different pyrocatechol concentrations for nine-layer LB films (Figure S2) (PDF)
