# Supplementary material for: Graphene Oxide and Conductive Polymer–Enhanced Langmuir–Blodgett Biosensor for Sensitive Detection of Pyrocatechol
Source: ACS Omega. 2026 Feb 16;11(8):13718–32. doi: 10.1021/acsomega.5c11698 (PMC12961474; doi:10.1021/acsomega.5c11698)
Supplement: Supplementary file 1 [file ao5c11698_si_001.pdf]

Graphene Oxide and Conductive Polymer–Enhanced Langmuir-Blodgett Biosensor for Sensitive Detection of Pyrocatechol

Felipe Merloto Marinho[1], Coral Salvo-Comino[2,3], Maria Luz Rodriguez-Mendez[2,3], José Roberto Siqueira Junior[4], Luciano Caseli[1,*]

[1]Hybrid Materials Laboratory, Department of Chemistry, Institute of Environmental, Chemical and Pharmaceutical Sciences, Federal University of São Paulo, Diadema, SP, Brazil, 09913-030

[2]Group UVASENS, Department of Inorganic Chemistry. Escuela de Ingenierías Industriales, University of Valladolid, Valladolid, Spain, 47011.

[3] BioecoUVA Research Institute, University of Valladolid, Valladolid, Spain, 47001

[4]Laboratory of Applied Nanomaterials and Nanostructures, Department of Physics, Institute of Exact and Natural Science and Education, Federal University of Triângulo Mineiro, Uberaba, MG, Brazil, 38.064-200

*** lcaseli@unifesp.br**

Supporting Information

Table S1: Transfer Ratio for Mix Y-LB films without or with laccase (0.5 mg/mL) and GO (0.5 mg/mL). Constant Surface pressure = 30 mN/m. Substrate = ITO glass. The first layer was produced from the upstroke of the solid support passing through the floating monolayer

Layer Number	Mix+Laccase	Mix+Laccase+GO
1	0.979	1.032
2	0.595	0.899
3	0.681	0.751
4	-0.947	-0.719
5	1.329	0.911
6	-1.244	-0.781
7	1.195	0.878
8	-1.301	-0.694
9	1.289	0.779

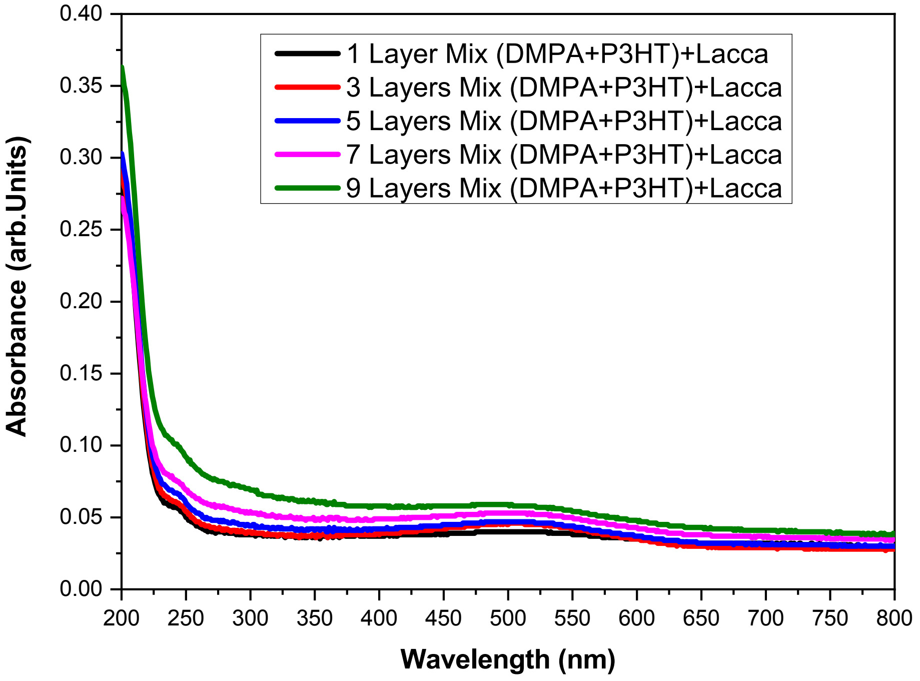

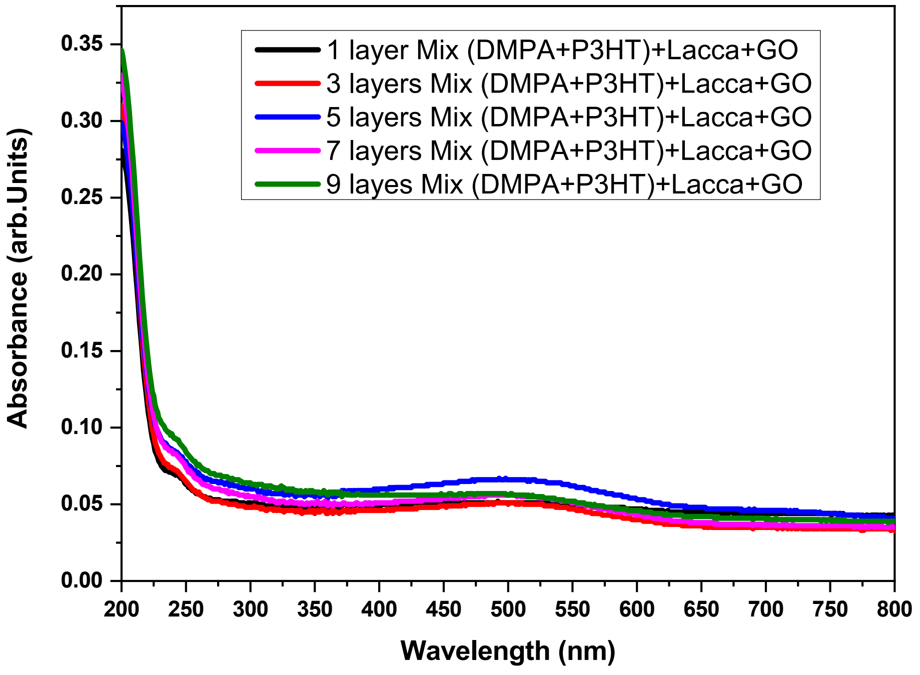

Figure S1: UV-vis spectra for the LB films (DMPA +P3HT + Laccase), without (top) or with (bottom) GO.

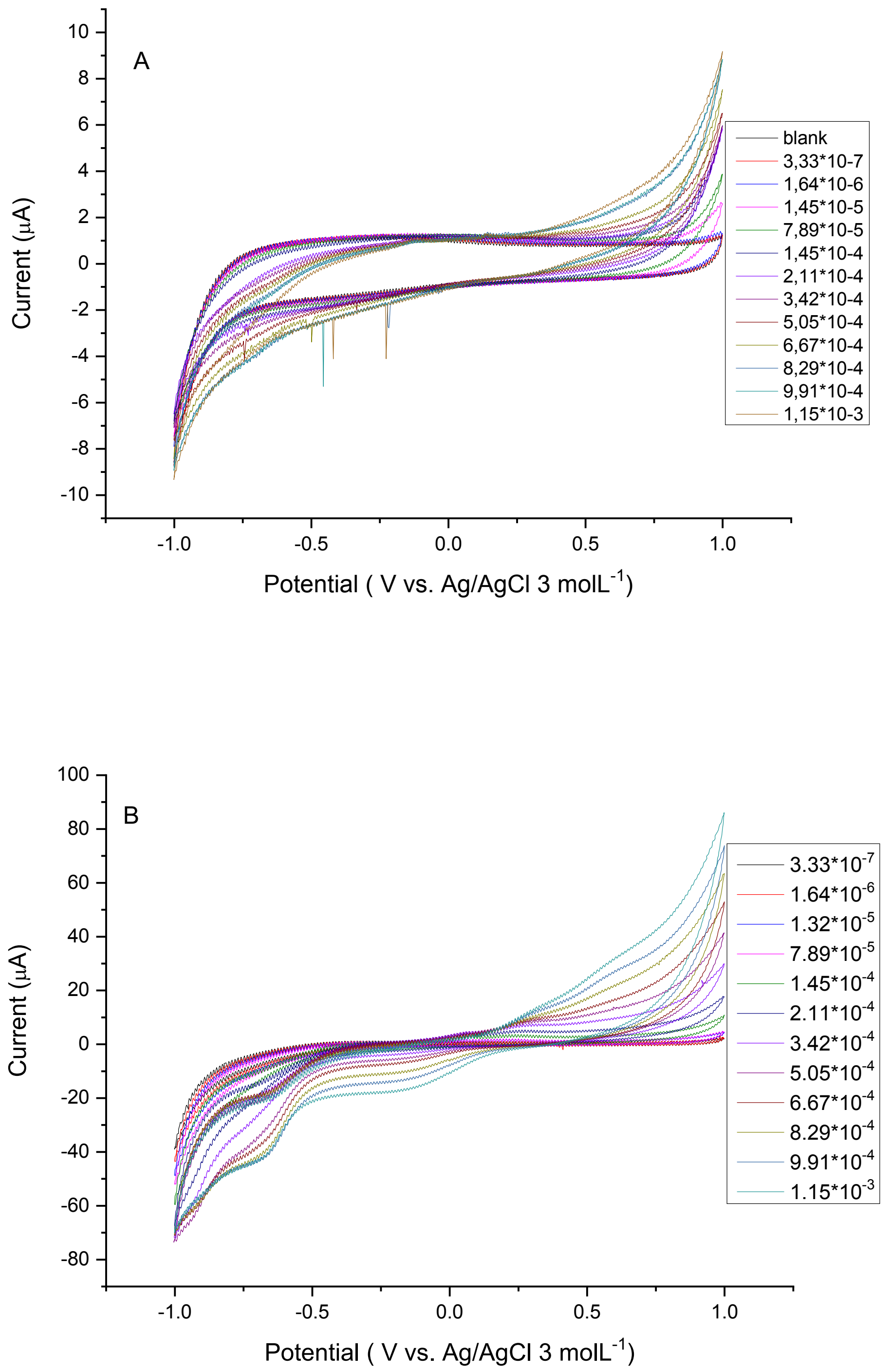

Figure S2: Cyclic voltammogram varying the pyrocatechol concentration from 3.33×10^{-3} to 1.15×10^{-3} mol.L^{-1}; (A) 9 layers Mix+Laccase as working electrode; (B) 9 layers Mix+Laccase+Graphene oxide as working electrode.
